# Morphology-based optical separation of subpopulations from a heterogeneous murine breast cancer cell line

**DOI:** 10.1371/journal.pone.0179372

**Published:** 2017-06-30

**Authors:** Masato Tamura, Shinji Sugiura, Toshiyuki Takagi, Taku Satoh, Kimio Sumaru, Toshiyuki Kanamori, Tomoko Okada, Hirofumi Matsui

**Affiliations:** 1 Biotechnology Research Institute for Drug Discovery, National Institute of Advanced Industrial Science and Technology (AIST), Tsukuba, Ibaraki, Japan; 2 Research Fellow of the Japan Society for the Promotion of Science, Tokyo, Japan; 3 Biomedical Research Institute, National Institute of Advanced Industrial Science and Technology (AIST), Tsukuba, Ibaraki, Japan; 4 Faculty of Medicine, University of Tsukuba, Tsukuba, Ibaraki, Japan; University of South Alabama Mitchell Cancer Institute, UNITED STATES

## Abstract

Understanding tumor heterogeneity is an urgent and unmet need in cancer research. In this study, we used a morphology-based optical cell separation process to classify a heterogeneous cancer cell population into characteristic subpopulations. To classify the cell subpopulations, we assessed their morphology in hydrogel, a three-dimensional culture environment that induces morphological changes according to the characteristics of the cells (i.e., growth, migration, and invasion). We encapsulated the murine breast cancer cell line 4T1E, as a heterogeneous population that includes highly metastatic cells, in click-crosslinkable and photodegradable gelatin hydrogels, which we developed previously. We observed morphological changes within 3 days of encapsulating the cells in the hydrogel. We separated the 4T1E cell population into colony- and granular-type cells by optical separation, in which local UV-induced degradation of the photodegradable hydrogel around the target cells enabled us to collect those cells. The obtained colony- and granular-type cells were evaluated *in vitro* by using a spheroid assay and *in vivo* by means of a tumor growth and metastasis assay. The spheroid assay showed that the colony-type cells formed compact spheroids in 2 days, whereas the granular-type cells did not form spheroids. The tumor growth assay in mice revealed that the granular-type cells exhibited lower tumor growth and a different metastasis behavior compared with the colony-type cells. These results suggest that morphology-based optical cell separation is a useful technique to classify a heterogeneous cancer cell population according to its cellular characteristics.

## Introduction

Most tumors are composed of different types of cells, including cancer cells, fibroblasts, vascular endothelial cells, and immune cells [1]. Furthermore, the population of cancer cells present in tumors exhibits remarkable variety with respect to clinically important phenotypes such as metastatic ability and chemotherapy resistance [2]. These heterogeneous phenotypes are thought to be related to heterogeneous genotypes, a disorganized microenvironment, and complex cellular networks; they are important in the development of next-generation cancer diagnostics and therapies [2,3]. However, limitations in experimental tools to classify these heterogeneous populations has hampered progress in analyzing and understanding tumor heterogeneity.

Cell separation should be a useful method for analyzing heterogeneous cell populations. Fluorescence-activated cell sorting (FACS) has been used to separate cells in suspension on the basis of their fluorescence color and intensity. Generally, proteins on the cellular membrane are labeled with fluorophore-conjugated antibodies and used as indicators for cell separation. FACS has been used to analyze tumor heterogeneity [4,5]; however, its application is limited to floating cells or cells retrieved from an adhesion culture. Furthermore, an appropriate surface marker is necessary to separate the cells, and such markers to separate heterogeneous tumor cell populations are often unavailable.

In contrast, three-dimensional (3D) cell culture in hydrogels is a general approach for biomimetic culture *in vitro* [6]. In a 3D culture environment, the composition and elasticity of the hydrogels significantly affect the growth and morphology of the cells [7]. Natural materials such as collagen, gelatin, fibrin, and Matrigel have been used as extracellular matrices for 3D cell cultures [8]. Of these materials, Matrigel is one of the most popular for the analysis of cancer cells in 3D cell cultures [9]. Matrigel comprises more than one thousand proteins including extracellular matrix proteins and growth factors [10], which regulate cellular activities [11]. Accordingly, Matrigel has been used widely in cancer research, including studies of angiogenesis and invasion, in multicellular spheroid assays, and in the preparation of xenograft models. Cancer cells exhibit characteristic behaviors in Matrigel-based 3D cultures (i.e. growth, invasion, and colony formation) [12]. In particular, the morphology of breast cancer cells in Matrigel differs depending on their malignant behavior and gene and protein expression profiles [13]. Therefore, morphology in hydrogel should be a useful indicator for classifying heterogeneous cancer cell populations.

In our previous studies, we synthesized photodegradable gelatin (PD-gelatin) hydrogels and established an optical cell separation system [14,15]. The hydrogels were prepared through a click reaction by simply mixing solutions of azide-modified gelatin (azide-gelatin) and the photocleavable cross-linker dibenzocycloctyl-terminated tetra-arm polyethylene glycol (DBCO-PC-4armPEG). The click reaction between the azide moiety and the DBCO moiety is biorthogonal; the reaction barely damages cells because the reactive moieties do not react with any compounds in the culture system including the culture medium or components of the cell membrane. Hydrogel formation via this click reaction occurs within 15 to 30 min of mixing [15]. Moreover, HeLa cell growth was enhanced by the addition of Matrigel to the PD-gelatin hydrogels [15]. Therefore, hydrogel encapsulation in PD-gelatin containing Matrigel should be an appropriate culture condition for separating cancer cells on the basis of their characteristics.

We previously used these 4T1E cells to establish highly bone marrow metastatic cells (4T1E/M3) through cycles of transplantation of 4T1E cells into mice and harvesting the metastatic tissue [16]. 4T1E cells were established by introducing neomycin resistance gene into 4T1 cells, which is a BALB/c-derived spontaneous mouse mammary carcinoma cell line, for *in vivo* selection [16–18]. Therefore, our 4T1E cell population should contain highly metastatic cells. We used optical cell separation to classify 4T1E cells into two types in a present study, one of which exhibited a colony-type morphology and the other a granular-type morphology in the PD-gelatin hydrogel containing Matrigel. We investigated the relationship between the morphology of the cells in the PD-gelatin hydrogel and their characteristics by assessing the cells in terms of spheroid formation *in vitro*, and tumor growth and metastasis *in vivo*.

## Materials and methods

### Materials

4T1E cells, which are the parent line of 4T1E/M3 cells, were established in our previous study [16]. RPMI-1640 supplemented with 4,500 mg/L glucose, L-glutamine, phenol red, HEPES, and sodium pyruvate was obtained from Wako Pure Chemical Industries, Ltd. (Tokyo, Japan) for culturing the 4T1E cells. Cell culture medium was prepared by adding 10 vol% heat-inactivated fetal bovine serum (HyClone Laboratories, Logan, UT, USA) and 1 vol% penicillin/streptomycin (Nacalai Tesque, Inc., Kyoto, Japan) to the RPMI-1640. Matrigel was obtained from Corning Inc. (Corning, NY, USA). SU-8 2075 negative photoresist was obtained from MicroChem (Newton, MA, USA). 1H,1H,2H,2H-Perfluorooctyltrichlorosilane was obtained from Wako Pure Chemical Industries, Ltd.

### Preparation of a sandwich culture chamber

A sandwich culture chamber to culture the cells between the two layers of the PD-gelatin hydrogel was prepared by means of soft lithography [19]. The chamber had a circular well with a diameter of 5.0 mm and a depth of 418 ± 10 μm, the internal volume of which was calculated to be 8.2 μL. A master template of the sandwich culture chamber was created by means of photolithography using SU-8 2075 negative photoresist. This process, including spin-coating, soft-baking, exposure, post-exposure baking, and development, was carried out according to the supplier’s information. The master template was treated with 1H,1H,2H,2H-perfluorooctyltrichlorosilane to reduce adhesive interaction with polydimethylsiloxane. Injection-molded polydimethylsiloxane produced by Meiho Co. Ltd. (Fukuoka, Japan) was used for the sandwich culture chamber.

### Optical cell separation using photodegradable hydrogels

The photodegradable hydrogels, azide-gelatin and DBCO-PC-4armPEG, were prepared as described previously [15]. Azide-gelatin prepared at a 100:25 molar ratio of the amino moiety in the gelatin to the azide moiety in the precursor compound was used in this study (25).

**Fig 1:**
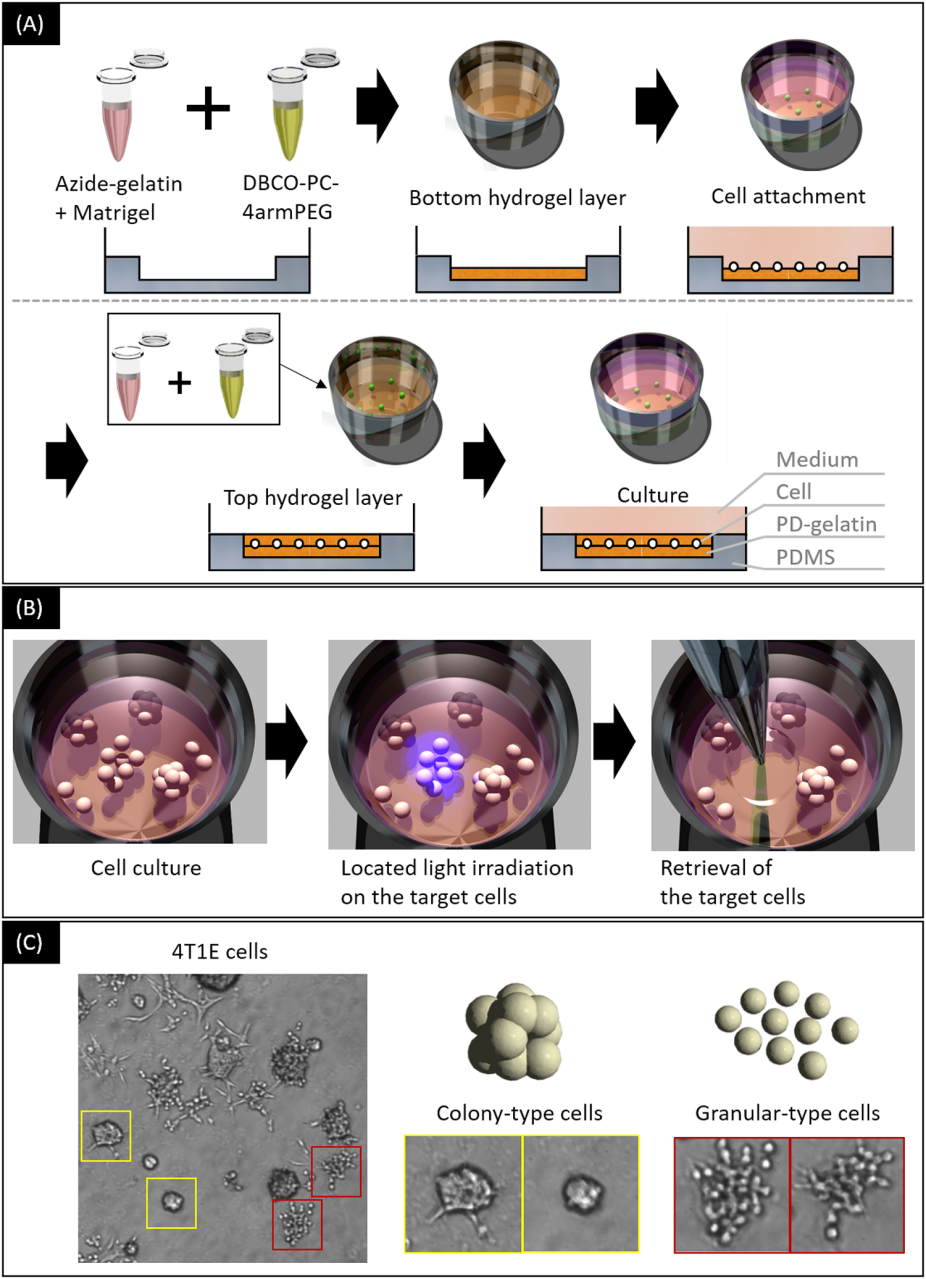
An overview of the optical cell separation procedure. (A) Cell encapsulation in the PD-gelatin hydrogel. 4T1E cells were encapsulated in photodegradable hydrogels prepared by mixing azide-gelatin solution and DBCO-PC-4armPEG solution. (B) Optical cell separation from the PD-gelatin hydrogel. The target cells are irradiated with UV light and subsequently retrieved after hydrogel degradation. (C) Microscopic images of 4T1E cells, and colony- and granular-type cells to be separated by use of the optical cell separation process.

Optical cell separation was conducted as described previously [14] (Fig 1A and 1B). We used a sandwich culture chamber to culture the cells in the PD-gelatin hydrogel containing Matrigel (Fig 1A). A solution of 25 mg/mL azide-gelatin (25) containing 1 mg/mL Matrigel and a solution of 1.2 mM DBCO-PC-4armPEG were prepared in culture medium. These solutions were mixed at 1:1 volume ratio by pipetting, then the mixture was poured into a well of the sandwich culture chamber as the bottom hydrogel layer. After a 30-min incubation at room temperature, cells suspended in culture medium were inoculated onto the bottom hydrogel layer at 51 cells/mm^2^. The cells were then incubated for 3 h at 37°C to allow them to adhere to the bottom hydrogel layer. The top hydrogel layer was prepared by using the same protocol as that used for the bottom hydrogel layer. The total height of the bottom and top hydrogel layers was calculated to be 200 μm (4 μL in 20 mm^2^). Three days after encapsulation, the 4T1E cells exhibited colony- and granular-type morphology (Fig 1C). Optical cell separation was performed within 14 days of cell inoculation. The colony- and granular-type cells were separated by using a maskless light irradiation apparatus (DESM-01, Engineering System, Co. Ltd., Matsumoto, Japan) [20], which enabled us to degrade only the PD-gelatin that surrounded the target cells. After the area surrounding the target cells was irradiated for 30 s with light with a wavelength of 365 nm, the sandwich culture chamber was incubated for 60 min at 37°C to degrade the PD-gelatin hydrogel. The target cells were collected by pipetting them in 500 μL culture medium, which was then transferred to a 48-well dish. The obtained cells were grown in 48-well dishes, 6-cm dishes, and 10-cm dishes as the number of cells increased; these cells were then used for characterization studies.

To observe cell morphology, the separated cells were cultured for 3 days in culture dish and in PD-gelatin hydrogels at 25 cells/mm^2^ using the sandwich culture chamber. Cell images in the culture dish (2D) and in the sandwich culture chamber (3D) were captured by using an inverted microscope (IX-71, Olympus Co., Tokyo, Japan).

### Spheroid assay

The 4T1E and separated cells were inoculated in 100 μL culture medium at 500 cells/well into a V-bottom non-adhesive 96-well plate (prime surface 96V plate, Sumitomo Bakelite Co., Ltd., Tokyo, Japan) to allow spheroid formation. Half volume of medium in each well was changed with fresh medium after three days. Images of the cellular aggregates were captured by using an inverted microscope after incubation periods of 24, 48, 72, and 144 h at 37°C. The projected area of cellular aggregate was measured by using ImageJ 1.45s software ver.1.6.0 (National Institutes of Health, Bethesda, MD, USA).

### Animal study

The animal study was approved by both the committee for experiments involving animals in AIST (No. 2016–212) and by an animal care and use committee at the University of Tsukuba (No. 16–163). Four-week-old CAnN.Cg-Foxn1nu/CrlCrlj (BALB/c-nu) male mice were purchased from Charles River Laboratories Japan, Inc. (Kanagawa, Japan). The 4T1E cells and the separated cell populations in 100 μL Hank’s balanced salt solution (Thermo Fisher Scientific K.K., Kanagawa, Japan) were transplanted by subcutaneous injection (10^6^ cells) into the left leg of mice. Four mice were used for each cell-type group (total twenty mice). The mice for each cell-type group were housed in a standard cage. Mice were allowed food and water ad libitum. We exchanged the bedding once a week. Tumor size and body weight were monitored every three to seven days after the injection. Statistical significance was analyzed by using Scheffe’s test on data from the mice that survived for the duration of the study period (KaleidaGraph 4.0, HULINKS Inc., Tokyo, Japan).

In the experiment, the time when the tumor exceeded 10% of the body weight was regarded as a humanitarian end point, the mouse was euthanized by cervical dislocation. Since the tumor forms a tumor mass on the surface when implanted subcutaneously, approximate the volume of 1 cm^3^ of the tumor mass was estimated as 1 g, and the tumor mass weight was compared with the body weight. In addition, when an unexpected situation occurs, the mouse had to be euthanized and the experiment had to be stopped as well. We were monitoring animal conditions every two to seven days. The health was monitored by observing the glossy coat, tremor, and activity, in addition to the body weight measurement. When all of the mice in one group showed signs of death or humane endpoint criteria, the experiment was terminated and all mice were euthanized. At this point, all mice were sacrificed by cervical dislocation, dissected, and their lung, liver, kidney, spleen, and intestine tissues were observed to study the frequency of metastasis at the endpoint. These humane endpoint criteria were approved by our animal ethics committee.

## Results

### Optical separation of 4T1E cells

**Fig 2:**
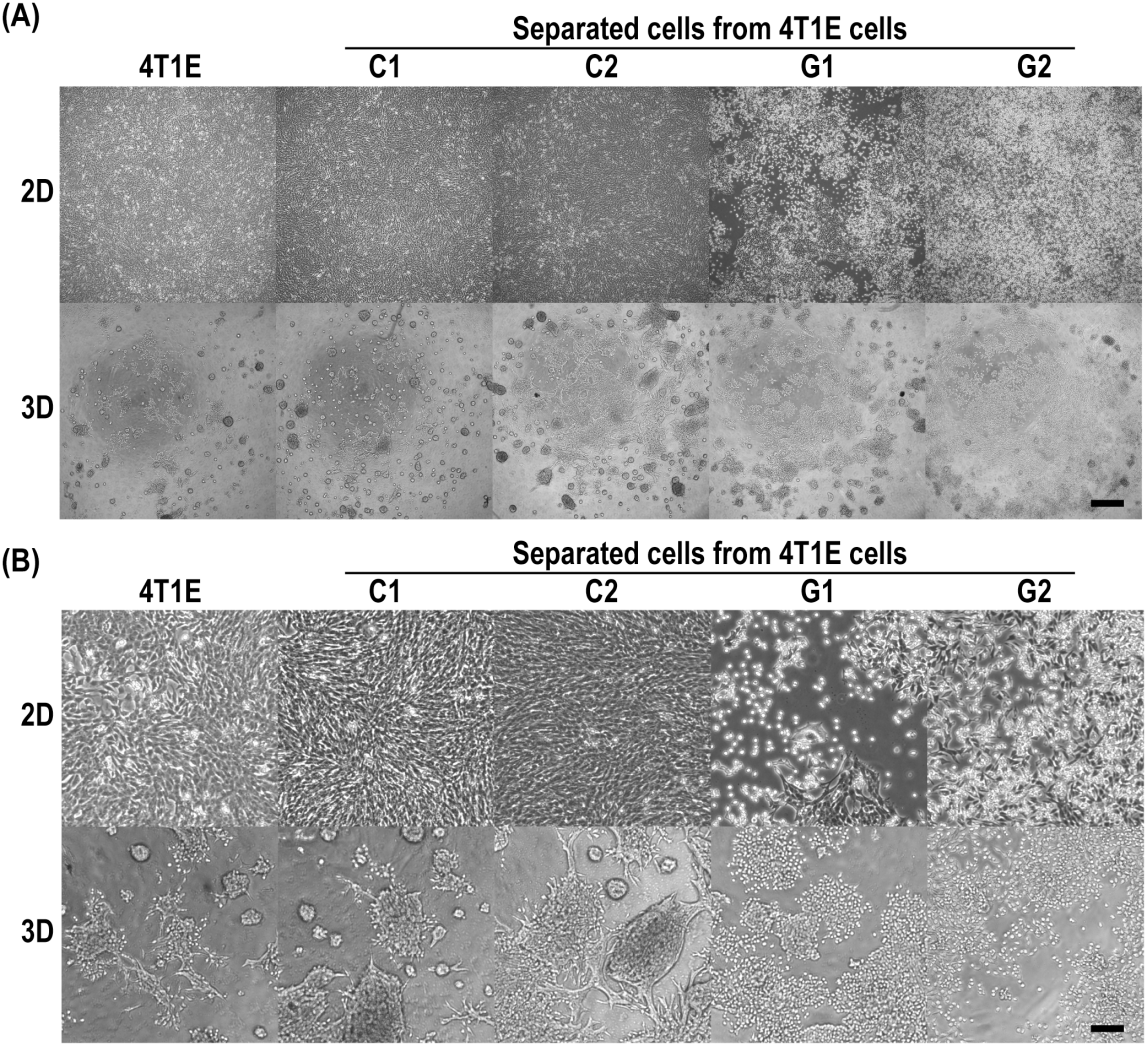
Microscopic images of 4T1E cells, and colony- and granular-type cells separated from 4T1E cells. The separated cells were established as colony- (C1 and C2) and granular-type (G1 and G2) cells by optical separation based on morphology in PD-gelatin hydrogels. 2D, cell culture on a culture dish; 3D, cell culture in the PD-gelatin hydrogels. The images were taken after 3 days of culture under 2D and 3D conditions. (A) Raw images. The scale bar indicates 500 μm. (B) Digitally magnified images. The scale bar indicates 125 μm.

We separated colony- and granular-type cells from the heterogeneous population of 4T1E cells by using optical cell separation. In so doing we generated five distinct cell populations for our analyses: 4T1E cells, C1 cells, C2 cells, G1 cells, and G2 cells. The separated colony- (C1 and C2) and granular-type (G1 and G2) cells were distinguished as same morphology-type, respectively. These cells exhibited different morphologies under 2D and 3D culture conditions (Fig 2). Under 2D culture conditions, the C1 and C2 cells attached to the surface of the culture dish strongly, whereas the G1 and G2 cells attached weakly. Under 3D culture conditions, the C1 and C2 cells exhibited colony-type morphology and the G1 and G2 cells showed granular-type morphology as was observed before optical cell separation. In addition, the C1 and C2 cells formed invadopodia in the PD-gelatin hydrogel (Fig 2B).

### Spheroid assay

**Fig 3:**
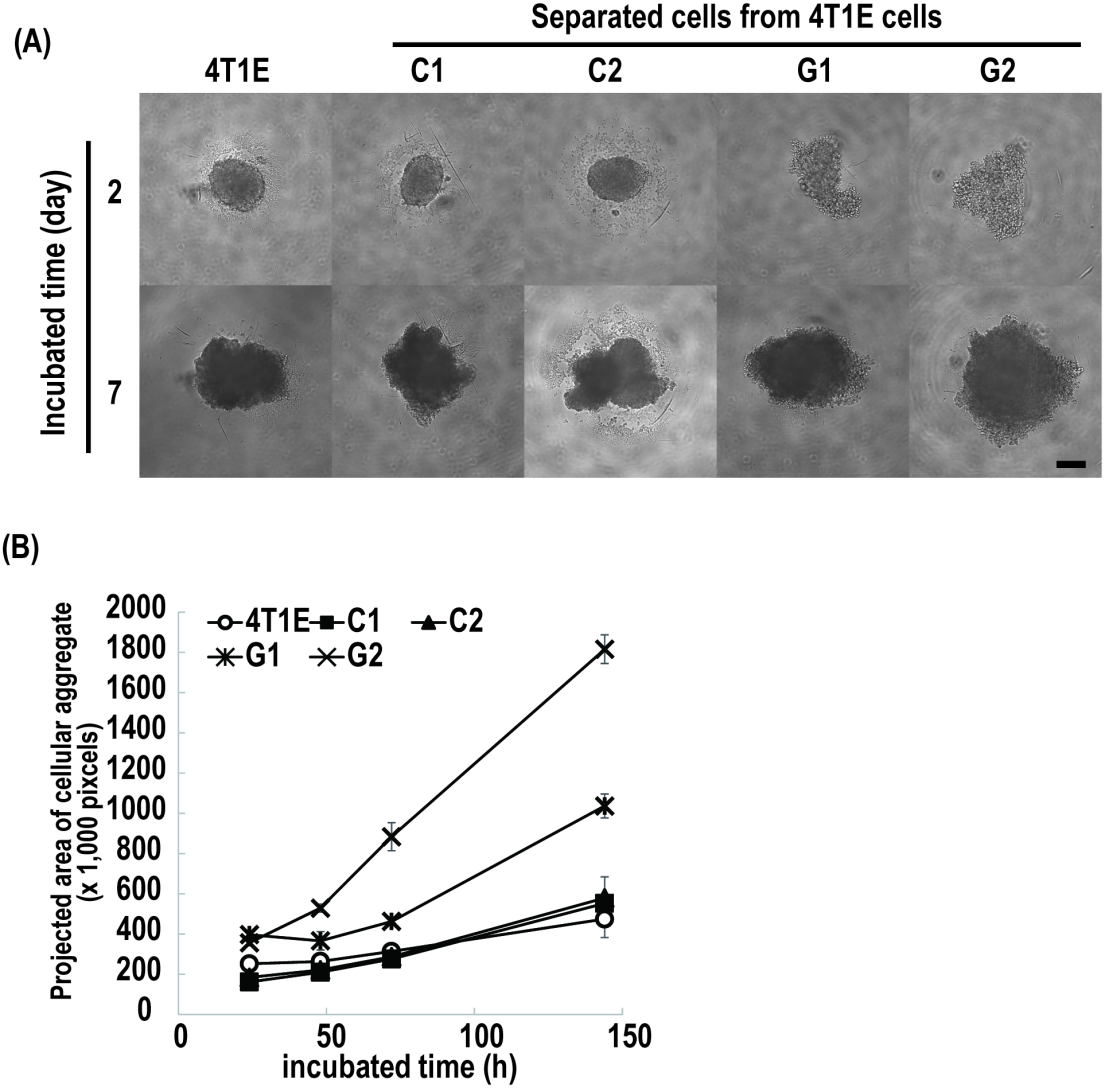
Spheroid assay for 4T1E cells, and colony- (C1 and C2) and granular-type (G1 and G2) cells separated from 4T1E cells. (A) Microscopic images of 4T1E cells, and colony- and granular-type cells cultured at 500 cells/well in a 96-well dish to assess spheroid formation. The scale bar indicates 200 μm. (B) Projected area of aggregates formed by 4T1E cells, and colony- and granular-type cells. Error bars indicate standard deviation.

The spheroid-forming ability of the 4T1E cells, and the separated colony- and granular-type cells was determined in a V-bottom non-adhesive 96-well plate as an estimate of tumorigenicity. The C1 and C2 cells formed spheroids in the wells within 2 days of culture, whereas the G1 and G2 cells spread out on the bottom of the well as they grew (Fig 3A). The projected area of cellular aggregates formed by the G1 and G2 cells was larger than that formed by the 4T1E cells, and that formed by the C1 and C2 cells (Fig 3B). These results indicated that the granular-type cells have stronger intracellular interaction to form compact spheroids than the granular-type cells.

### Tumor growth and metastasis

**Fig 4:**
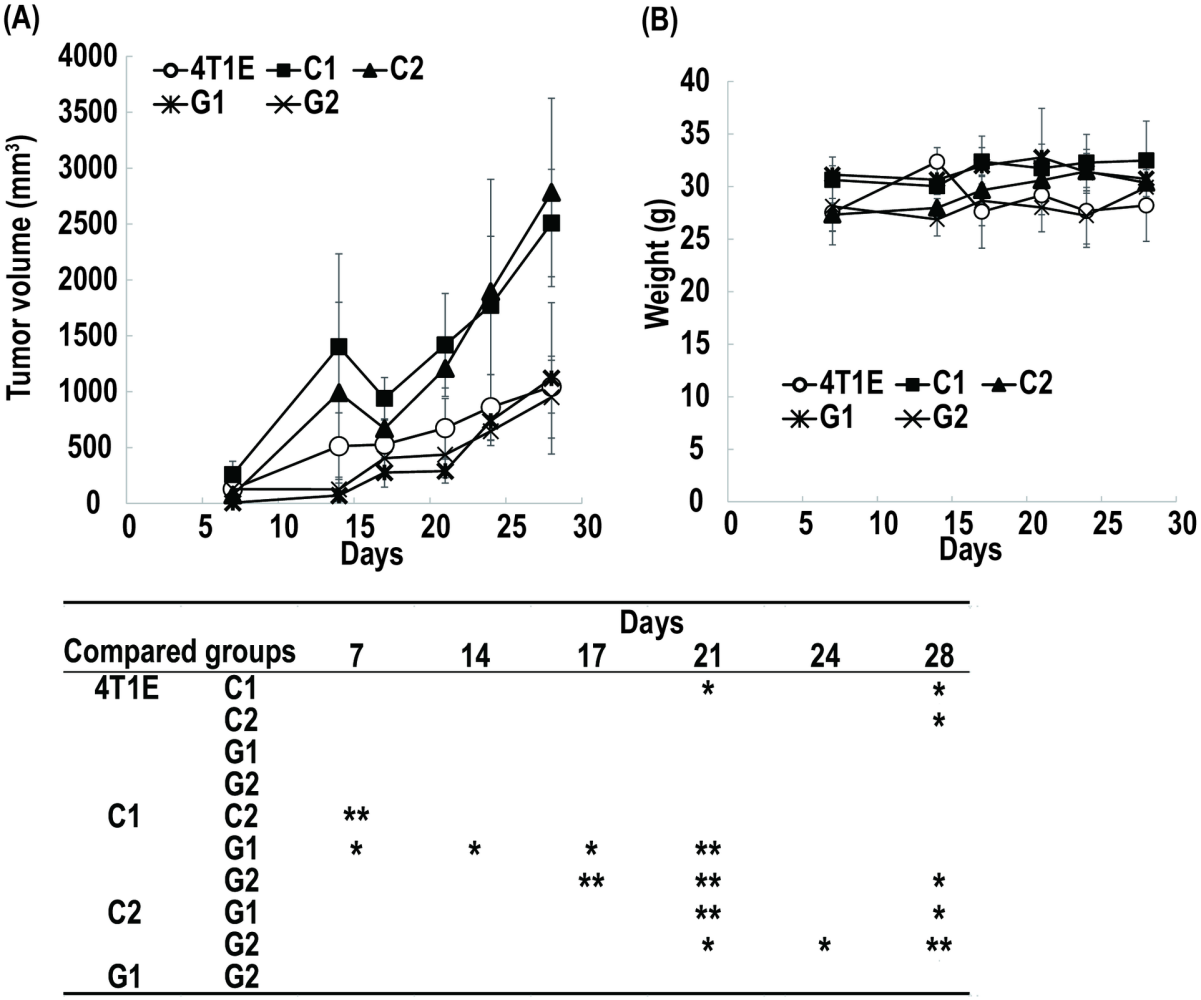
Tumor growth in mice transplanted with 4T1E cells, and colony- (C1 and C2) and granular-type (G1 and G2) cells separated from 4T1E cells. (A) Tumor growth. (B) Body weight of mice. The table below the figure shows the statistical profile as analyzed by using Scheffe’s test for tumor growth. Asterisks in the table indicate statistical significance. One asterisk indicates *p*-value less than 0.05 and double asterisks indicate *p*-value less than 0.01. Error bars indicate the standard deviation (n = 3–4).

**Fig 5:**
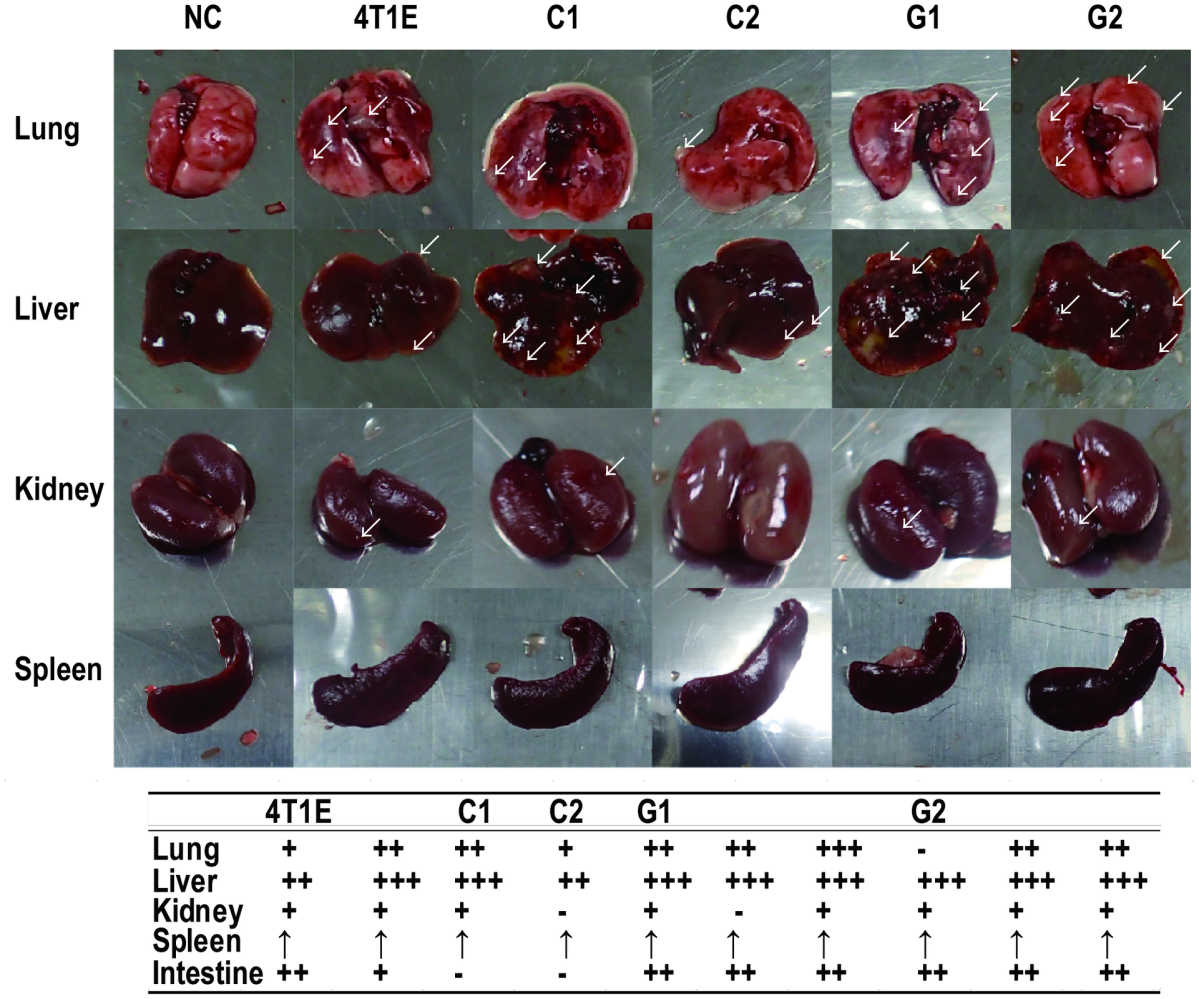
Frequency of metastasis in tissues obtained 38 days post-transplantation of 4T1E cells, and colony- (C1 and C2) and granular-type (G1 and G2) cells separated from 4T1E cells. Arrows indicate metastatic tumors on the surface of the tissues. The table shows the frequency of metastases confirmed by observation of the dissected tissues. The number of tumors detected in lung, liver, colon, kidney, and intestine is indicated as none (-), one (+), one to five (++), and more than five (+++). The upward-pointing arrows in the table indicate spleen enlargement.

Tumor growth and metastasis of the 4T1E cells, and the separated colony-, and granular-type cells were evaluated after transplantation of the cells into mice. The tumor sizes of the colony-type cells (C1 and C2) increased more quickly after transplantation than did those of the 4T1E cells or those of the granular-type (G1 and G2) cells (Fig 4A). We did not detect the statistically significant difference in the mouse body weight throughout the experimental period between each study group (Fig 4B). One mouse transplanted with G1 cells was dead after 28 days without meeting criteria for euthanasia. One mouse transplanted with C1 cells and three mice transplanted with C2 cells were met the criteria for euthanasia and euthanized by cervical dislocation after 31 days. Two, two, and one mice transplanted with 4T1E cells, C1 cells, and G2 cells, respectively, were dead without meeting the criteria after 38 days. The cause of death of these mice without meeting criteria for euthanasia was unknown. At the time, two, one, one, three, and three mice transplanted with 4T1E, C1, C2, G1, and G2 cells, respectively, were alive. Additionally, one mouse transplanted with the 4T1E cells and all mice transplanted with C1 or C2 cells met the criteria for euthanasia, and other mice showed that its health condition had more deteriorated. We considered an animal study endpoint, we dissected the mice after 38 days post-transplantation for studying the frequency of metastasis. We observed metastases in various tissues, including lung, liver, kidney, and intestine, of mice that had been transplanted with the granular-type cells (G1 and G2) as the mice that had been transplanted with the 4T1E cells (Fig 5; tissue images are shown in S1 Fig). On the other hand we did not observe metastases in intestine of mice that had been transplanted with the colony-type cells (C1 and C2) (Fig 5 and S1 Fig). Overall, we observed formation of larger tumors in mice transplanted with the colony-type cells (C1 and C2) and relatively frequent metastasis in mice transplanted with the granular-type cells (G1 and G2).

## Discussion

In this study, we separated colony-type (C1 and C2) and granular-type (G1 and G2) cells from the heterogeneous murine breast cancer cell line 4T1E by using an optical cell separation technique. The separated colony- and granular-type cells exhibited different characteristics in terms of spheroid formation *in vitro* and tumor growth and metastasis behavior *in vivo*. Our findings suggest that cellular morphology in PD-gelatin hydrogel containing Matrigel could be used to classify cells in a heterogeneous cancer cell population on the basis of the characteristics of the cells. Optical cell separation using PD-gelatin hydrogel containing Matrigel could therefore be a useful method to classify cells in a heterogeneous cancer cell population. A cancer cell population organized into subpopulations by using such a method would likely have better-defined property characteristics than the parent heterogeneous population. Such a classified cancer cell population could be a source of novel cancer cell lines that would be of value for basic cancer research.

4T1E and 4T1E/M3 cells have been shown to exhibit spheroidal growth in soft agar [16]. In the present study, we observed that colony-type (C1 and C2) cells formed invadopodia in the PD-gelatin hydrogel, whereas granular-type (G1 and G2) cells exhibited less interaction with the PD-gelatin hydrogel matrix (Fig 2). The contrasting behaviors of the cells in the PD-gelatin hydrogel demonstrate the suitability of the PD-gelatin hydrogel as a material for characterizing cancer cells on the basis of their morphology in the hydrogel. In addition, we know that the microenvironment influences tumorigenicity, and that stromal factors facilitate tumor growth and angiogenesis [21]. Matrigel has been shown to be effective in culture conditions designed to reconstruct the biomimetic environment [22]. Therefore, 3D culture conditions that include Matrigel should be useful to characterize heterogeneous cancer cell populations. However, cellular shape is clearly affected by many parameters including nutritional status, culture media contents, and cell-matrix adhesions [23]. Therefore, optimization of the cell culture conditions for each cancer cell population would be required for each application, including cell separation for diagnostics or the establishment of a novel cancer cell line.

In this study, we distinguished cellular shape by observing the cells under the microscope. However, automated image analysis would afford better reproducibility and sensitivity to the classification, as reported for 2D culture conditions [24]. In the future, automated image analysis of 3D cultures should be developed for industrial applications of optical cell separation systems.

We previously reported that were able to establish highly metastatic 4T1E/M3 cells from 4T1E cells, which we used in this study [16]. The 4T1E/M3 cells exhibited spheroidal morphology in soft agar and were highly metastatic in mice. In contrast, the colony-type cells separated in this study exhibited spheroidal morphology in a 96-well plate but were partially metastatic in mice, whereas the granular-type cells exhibited non-spheroidal morphology in the 96-well plate but were metastatic in mice. Thus, the characteristics of the obtained colony- and granular-type cells differed from those of 4T1E/M3 cells. The molecular mechanism responsible for these different behaviors should be determined. It has been reported that the expression of intercellular adhesion molecule-1 (ICAM-1) is upregulated on 4T1E/M3 cells relative to that in 4T1E cells [16]. ICAM-1 is involved in anchorage-independent growth and migration activity, and its suppression inhibits human breast cancer cell invasion [25]. Therefore, the expression of ICAM-1 in colony- and granular-type cells is of interest. The expression of some molecular would be similar depending on morphology-type because drug response of tamoxifen in pilot study were clearly different between colony-type cells and granular-type cells (S2 Fig). We plan to analyze it in future experiments.

## Conclusions

In conclusion, the present study demonstrated the morphology-based optical cell separation of subpopulations from a heterogeneous cancer cell population in PD-gelatin hydrogels. The separated cells exhibited different phenotypes (tumorigenicity and frequency of metastasis) depending on their morphology in the PD-gelatin hydrogel. We believe that this morphology-based optical cell separation method would be useful for establishing various malignant cell lines from heterogeneous cancer cell populations and for developing a novel cancer diagnosis process that takes into account tumor heterogeneity.

## Supporting information

S1 Fig Images of tissues taken from mice transplanted with 4T1E cells or separated colony- (C1 and C2), or granular-type (G1 and G2) cells. Note, the spleens from the mice that received transplanted cells were enlarged. NC, normal control.
(TIF)

S2 Fig Drug response of tamoxifen with 4T1E cells or separated colony- (C1 and C2), or granular-type (G1 and G2) cells. Cells were cultured at 10^4^ cells/well in a 96 well plate for 3h, and then culture medium were changed tamoxifen-containing medium at 0, 10, 20, 50, 100, and 200 μM. After a drug exposure for 48 h, the medium was replaced to 10 v% WST reagent (Dojindo, Kumamoto, Japan)-containing culture medium, and incubated for 1 h. The absorbance at 450 nm wavelength was measured by a multimode plate reader (DTX 880, Beckman Coulter, CA, USA).
(TIF)
