# Supplementary figures and images for: Morphology-based optical separation of subpopulations from a heterogeneous murine breast cancer cell line
Source: PLoS One. 2017 Jun 30;12(6):e0179372. doi: 10.1371/journal.pone.0179372 (PMC5493304; doi:10.1371/journal.pone.0179372)

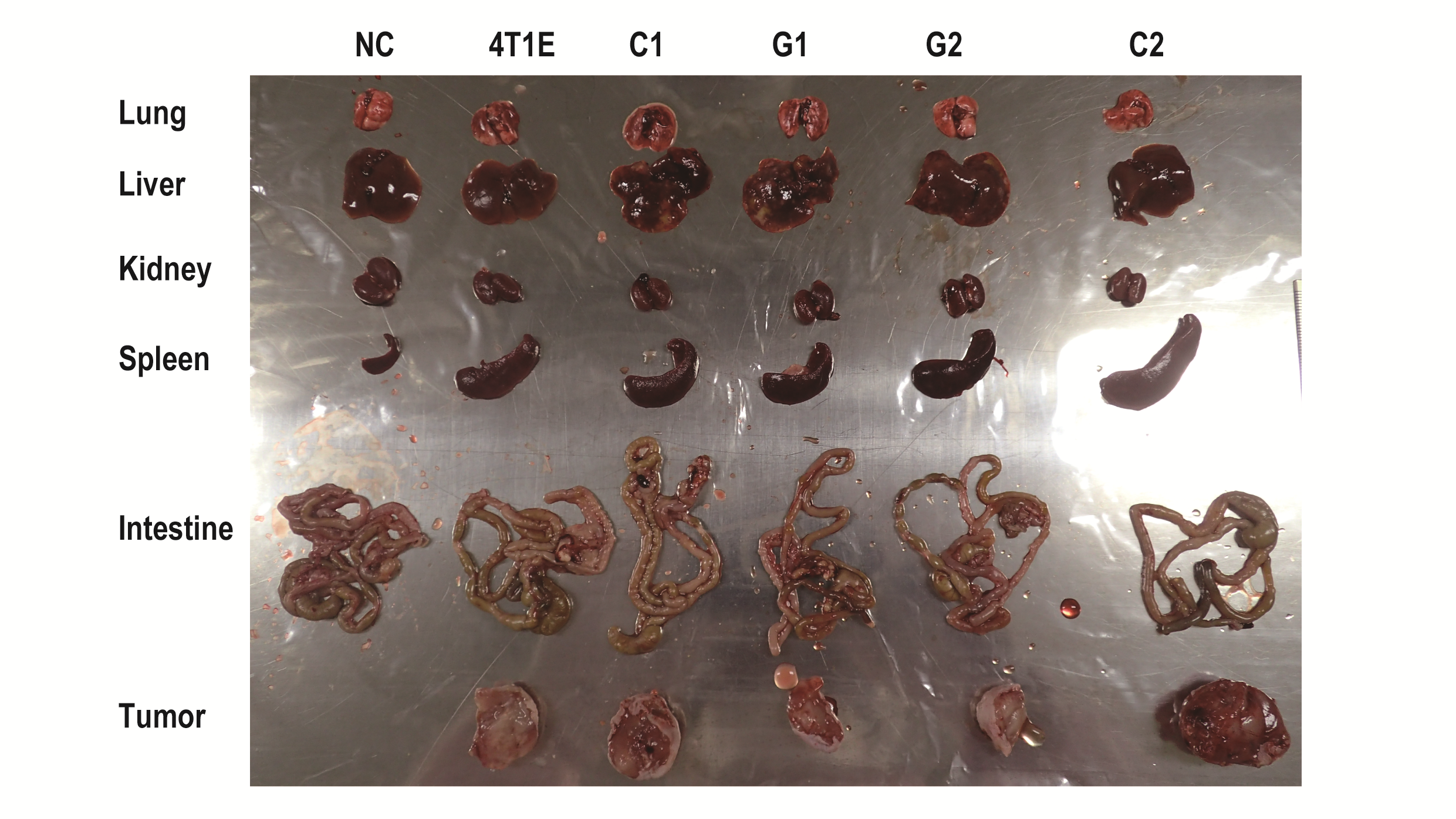

Supplement: S1 Fig — Note, the spleens from the mice that received transplanted cells were enlarged. NC, normal control. (TIF) [file pone.0179372.s001.tif]

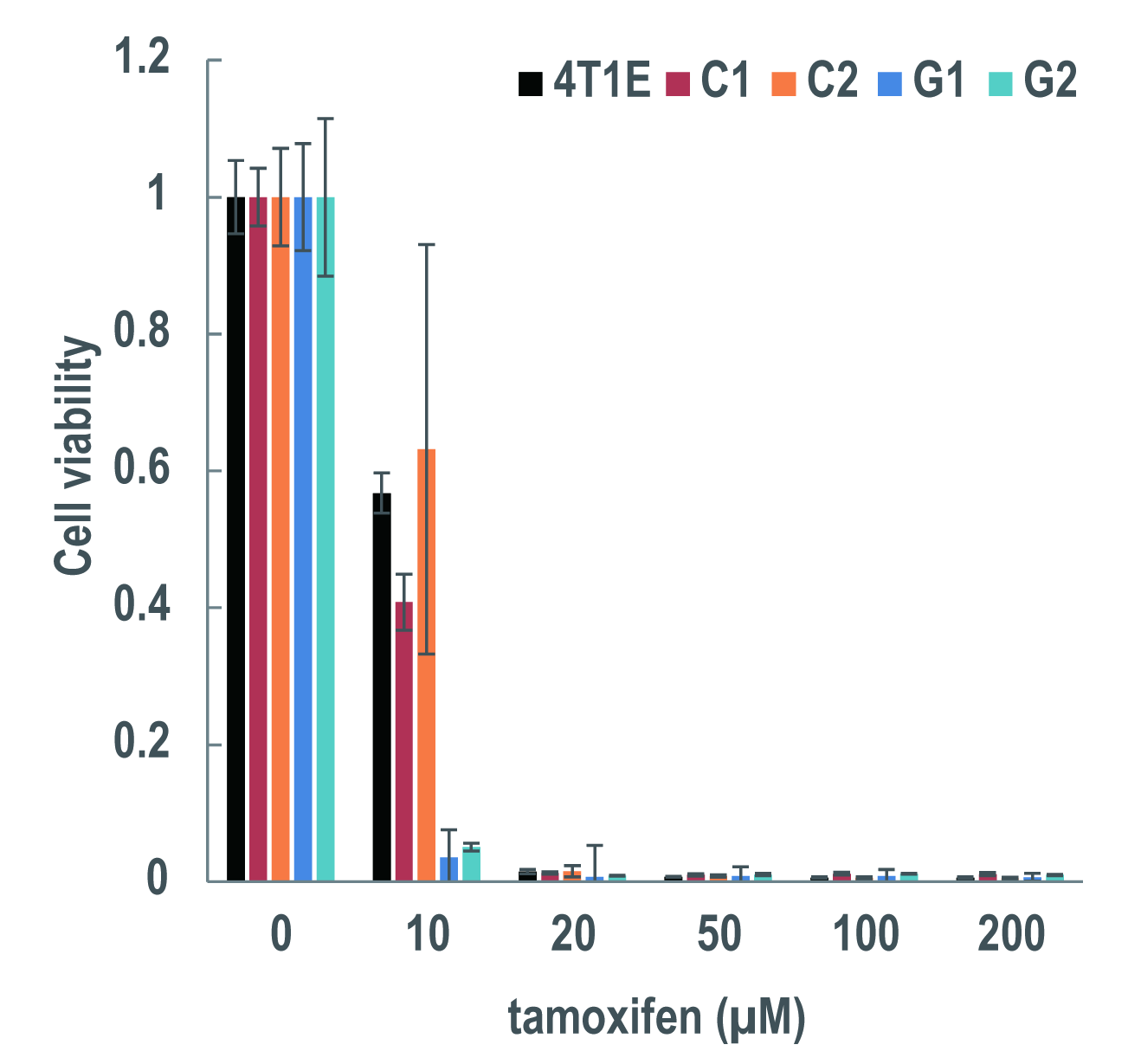

Supplement: S2 Fig — Cells were cultured at 104 cells/well in a 96 well plate for 3h, and then culture medium were changed tamoxifen-containing medium at 0, 10, 20, 50, 100, and 200 μM. After a drug exposure for 48 h, the medium was replaced to 10 v% WST reagent (Dojindo, Kumamoto, Japan)-containing culture medium, and incubated for 1 h. The absorbance at 450 nm wavelength was measured by a multimode plate reader (DTX 880, Beckman Coulter, CA, USA). (TIF) [file pone.0179372.s002.tif]
